# Investigation of Trunk and Pelvis Muscle Activity during Sprinting using T2-Weighted Magnetic Resonance Imaging

**DOI:** 10.5114/jhk/197315

**Published:** 2025-05-29

**Authors:** Takaya Yoshimoto, Yoshihiro Chiba, Hayato Ohnuma, Norihide Sugisaki

**Affiliations:** 1 Faculty of Welfare Society, The International University of Kagoshima, Kagoshima, Japan.; 2 Department of Sports Sciences, Japan Institute of Sports Sciences, Tokyo, Japan.; 3 Faculty of Management, Josai University, Saitama, Japan.; 4 Faculty of Health and Welfare, Kobe Women’s University, Hyogo, Japan.; 5 Center for Liberal Arts, Meiji Gakuin University, Tokyo, Japan.

**Keywords:** erector spinae, gluteus maximus, psoas major, lateral abdominals, pectineus

## Abstract

There are few studies that clarify the level of muscle activity in the trunk and pelvis muscles during sprinting. This study aimed to investigate muscle activity in the trunk and pelvis muscles during sprinting using T2-weighted magnetic resonance imaging (MRI). The pre- and post-test designs were employed by measuring trunk and pelvis muscle activity using T2-weighted MRI before and after 60-m round-trip sprints. Ten male sprinters (N = 10, age, 23.3 ± 6.7 years; body height, 175.1 ± 3.6 cm; body mass, 66.8 ± 4.3 kg; 100-m personal record, 11.18 ± 0.48 s, means ± standard deviations [SDs]) performed three sets of three 60-m round-trip sprints. Before and after the round-trip sprints, 3T MRI scans were performed to obtain the T2 values of the trunk and pelvis muscles. After the 60-m roundtrip sprints, the T2 values of lateral abdominal, psoas major, erector spinae, gluteus maximus, gluteus medius, rectus femoris, tensor fasciae latae, sartorius and pectineus muscles increased significantly. There were intermuscular differences in the rate of change of T2 values before and after the 60-m round-trip sprints, with significantly higher levels of muscle activity in lateral abdominals, psoas major, erector spinae, gluteus maximus, and pectineus. In sprinting, the trunk and pelvis muscles were found to be specifically activated.

## Introduction

Physical movements result from a combination of joint torque generated by muscle contractions. Therefore, the critical muscles involved in movement must be identified when designing a training program to enhance physical performance. The critical muscles involved in sprinting have been investigated through morphometric measurements of a sprinter’s muscles using ultrasonography (Kumagai et al., 2000) and magnetic resonance imaging (MRI) (Bellinger et al., 2021; Ema et al., 2018; Hoshikawa et al., 2006; Sugisaki et al., 2018). This is because the force- or torque-generating capacity of a muscle is associated with its cross-sectional area or volume (Fukunaga et al., 2001). Previous research (Handsfield et al., 2017) has revealed differences in muscle volume in the lower extremity muscles, such as the semitendinosus and gracilis, between non-sprinters and sprinters. A significant relationship between sprint performance and the size of major lower limb muscles, such as the psoas major, the gluteus maximus, and the rectus femoris, has also been reported (Copaver et al., 2012; Ema et al., 2018; Hoshikawa et al., 2006; Tottori et al., 2021). However, the critical muscles predicted from muscle size are inconsistent across previous studies (Ema et al., 2018; Sugisaki et al., 2018; Tottori et al., 2021). Furthermore, the relationship between critical muscle size and sprint performance is weak (R2 = 0.234–0.643) (Ema et al., 2018; Sugisaki et al., 2018; Tottori et al., 2018). Therefore, morphological information alone cannot identify critical muscles in sprinting, and other vital factors should be considered.

The force generated by a muscle is associated with the muscle activity level (Akagi et al., 2009; Fukunaga et al., 2001; Ikai and Fukunaga, 1968). Therefore, the activity level of each muscle during sprinting is required to identify the critical muscles involved. Previous studies have quantified muscle activity levels during sprinting using surface electromyography (EMG) (Higashihara et al., 2018; Mero and Komi, 1994). Recently, T2-weighted MRI has been used to quantify muscle activity levels; it can assess the activity of deeper and superficial muscles (Bourne et al., 2021) and eliminate problems in EMG measurements, such as a shift in the electrodes’ position relative to the muscle fibers (Farina, 2006; Farina et al., 2004). Using this method, it was found that the gluteus maximus and semitendinosus exhibited particularly high muscle activity during sprinting, whereas the quadriceps exhibited no significant activity (Yoshimoto et al., 2022). These results were inconsistent with the findings of morphological studies on critical muscles (Ema et al., 2018; Tottori et al., 2018), further emphasizing the need to determine muscle activity levels during sprinting when investigating the critical muscles involved.

Assessment of muscle activity during sprinting using T2-weighted MRI has primarily focused on the lower extremity muscles. Currently, there is no information regarding the activity of trunk and pelvis muscles despite their reported importance (Rivera, 2016), mainly because the trunk and pelvis contain many deep muscles, making it difficult to comprehensively evaluate their activity during sprinting. Considering that the pelvis contains muscles involved in hip flexion and extension (Andersson et al., 1995; Copaver et al., 2012; Ema et al., 2018; Sugisaki et al., 2018) and that the trunk muscles also stabilize the trunk and contribute to lower limb muscle strength (Behm and Anderson, 2006; Behm et al., 2009; Butcher et al., 2007), it is expected that there are muscles located within the trunk and the pelvis that are highly activated and crucial in sprinting. Therefore, a thorough study of trunk and pelvis muscle activity, including deep muscles, could help identify specific muscles that require training to improve sprinting performance.

In this study, we used T2-weighted MRI to quantify the activity levels of most trunk and pelvis muscles during sprinting. The aim was to identify critical trunk and hip flexor muscle candidates for sprinting.

## Methods

### Participants

Ten male sprinters (N = 10; 100-m personal record, 11.18 ± 0.48 s, age, 23.3 ± 6.7 years; body height, 175.1 ± 3.6 cm; body mass, 66.8 ± 4.3 kg; means ± standard deviations [SDs]) participated in the study. Notably, all participants were free from cardiovascular, metabolic, and immunological disorders as well as orthopedic abnormalities and were not taking any medications that could affect muscle function. Participants had been competing in 100-m races for over four years and were engaged in specific sprint training programs for at least 3 h daily and 5 days weekly under the supervision of a coach. The study was approved by the Ethics Committee of the Japan Institute of Sports Sciences (protocol code: 002; approval date: 25 July 2018) and followed the guidelines for human experimentation. Before participation, sprinters were fully informed about the study’s procedures and potential risks, and written informed consent was obtained. However, to prevent bias, the study’s aims and hypotheses were not disclosed to participants until all measurements were completed.

### Design and Procedures

T2-weighted MRI was performed before and after repeated sprint exercises. Furthermore, MRI images were obtained following a thorough 1-h warm-up session to mitigate the risk of injury from sprinting immediately after rest. The initial half of the warm-up consisted of dynamic exercises to prepare the participants for maximal sprinting. However, the latter half included low-intensity exercises and stretching to minimize the metabolic impact. The sprinting distance was set at 60 m based on the finding that sprinters achieve their highest velocity between the 50^th^ and the 60^th^ m (Mackala, 2007). We hypothesized that lactate concentration would be minimal due to the short duration (6–7 s) of the 60-m sprint, which is insufficient to significantly alter T2 values. High lactate levels are typically observed during intermittent exercise involving alternating periods of activity and rest (Yoshimoto et al., 2022). Based on these insights, a 60-m round-trip sprint was selected to achieve peak running velocity and adequate lactate concentration. Participants performed three sets of three 60-m sprints from a standing start on an indoor track, with 30-s rest intervals between sprints and 3-min rest periods between sets. Sprint times were recorded using phototubes (Brower Timing System, USA) positioned at 0 and 60 m. Immediately after the sprints, participants were quickly transported to the MRI room within 1 min 30 s, and MRI scans were conducted within 5 min and 30 s post-sprint.

### Transverse Relaxation Time (T2)

MRI was conducted to measure muscle T2 relaxation time. A 3T MR system (MAGNETOM Verio; Siemens Healthineers, Erlangen, Germany) was used to capture images of the trunk and the pelvis using body and spine array coils. The imaging protocol involved a spin-echo pulse sequence with the following variables: repetition time of 2000 ms, echo times of 20/30/40/50/60/70 ms, slice thickness of 5 mm, matrix size of 256 × 256, one excitation, and total acquisition time of 5 min 37 s. Multiple slices in the imaging range were used to identify each muscle region of interest (ROI), and the ROI was created along the muscle shape. T2 values were measured using ImageJ software (Version 1.51k, National Institutes of Health, Bethesda, MD, USA). A priori, the measurer traced 84 muscles in the imaging range three times each and calculated the intraclass correlation coefficient. The resulting Cronbach’s alpha was 0.988, and values obtained from a single trace were used because of the high reproducibility of the measures. The muscles examined were the rectus abdominis, lateral abdominal muscles (abdominal obliques and transversus), psoas major, iliac, erector spinae, gluteus maximus, gluteus medius, gluteus minimus, obturator internus, piriformis, rectus femoris, tensor fasciae latae, sartorius, and pectineus muscles. We carefully excluded non-muscle areas, such as intramuscular fat and fascia, and secured the abdominal area with a corset during imaging to minimize breathing artifacts.

### Statistical Analysis

Descriptive statistics are reported as mean ± standard deviation (SD). We analyzed changes in T2 values for each muscle before and after 60-m round-trip sprints using a paired sample Student’s *t*-test. The threshold for statistical significance was set at *p* < 0.004 (0.05/14) using the Bonferroni correction for the paired *t*-test. Effect sizes were calculated using Cohen’s *d* and classified as very small (*d* = 0.01), small (*d* = 0.20), medium (*d* = 0.50), large (*d* = 0.80), very large (*d* = 1.20), and huge (*d* = 2.00) based on the guidelines provided by previous studies (Hopkins et al., 2009; Sawilowsky, 2009). All statistical data were analyzed using SPSS version 26.0 for Windows (IBM Japan). Statistical significance was set at *p* < 0.05.

## Results

Table 1 shows changes in sprint time for each 60-m round-trip. The fastest running time was in the first sprint of the first set and it was the slowest in the third sprint of the third set.

**Table 1:** Time of 60-m round-trip sprints.

	1^st^		2^nd^		3^rd^		total	
1 set [s]	7.42	(0.31)	7.61	(0.30)	7.90	(0.39)	22.93	(0.91)
2 set [s]	7.50	(0.38)	7.92	(0.43)	8.27	(0.63)	23.68	(1.24)
3 set [s]	7.73	(0.41)	8.19	(0.42)	8.40	(0.45)	24.32	(1.21)

**Figure 1:**
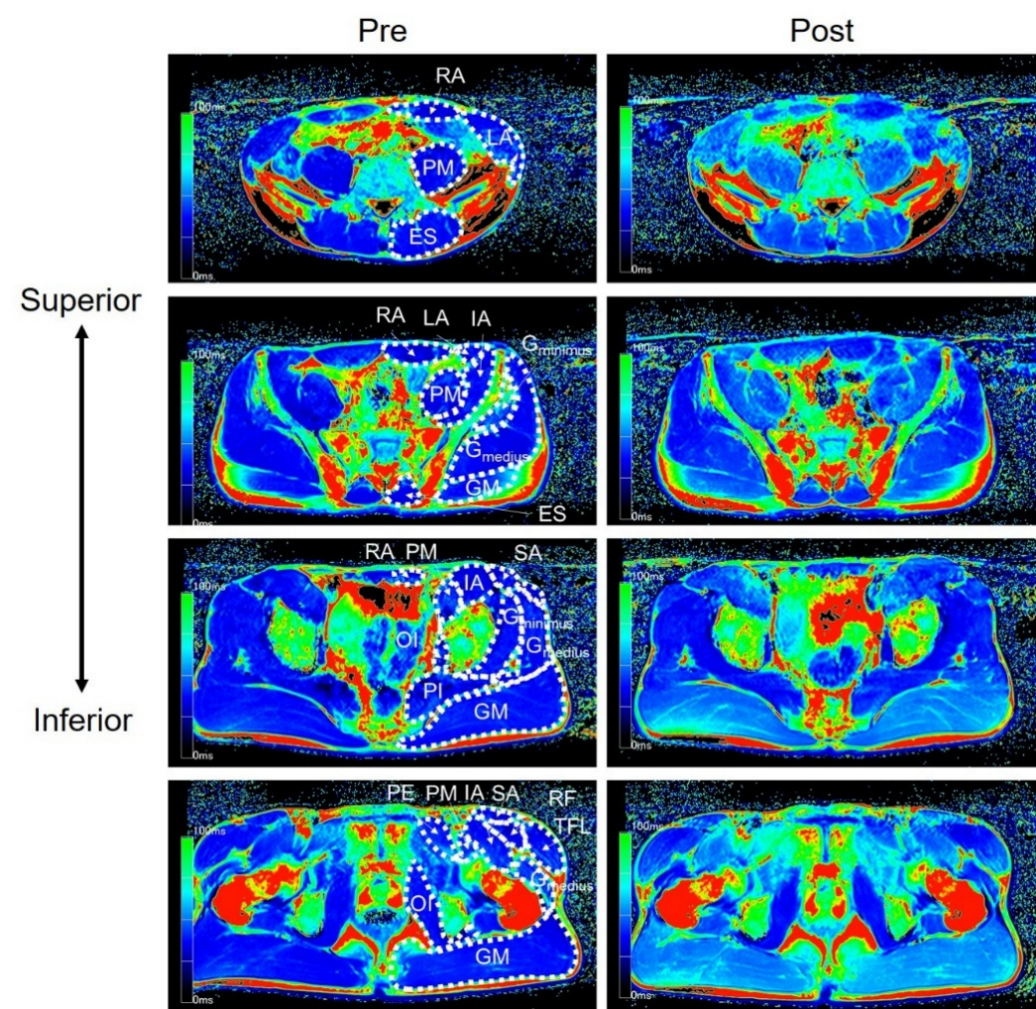
Examples of T2-weighted magnetic resonance images of the trunk and the pelvis. RA, rectus abdominis; LA, lateral abdominals; PM, psoas major; ES, erector spinae; IL, iliac; GM, gluteus maximus; G_medius_, gluteus medius; G_minimus_, gluteus minimus; PI, piriformis; SA, sartorius; TFL, tensor fasciae latae; RF, rectus femoris; PE, pectineus

Figure 1 shows a typical example of T2-weighted images before and after a 60-m round-trip sprint. T2 values increased significantly after a 60-m round-trip sprint in the lateral abdominal, psoas major, erector spinae, gluteus maximus, gluteus medius, rectus femoris, tensor fasciae latae, sartorius, and pectineus muscles (*p* < 0.004, Table 2). The effect size of particular muscles was medium to huge (*d* = 0.55–3.52, Table 2).

**Table 2:** Transverse relaxation time (T2) of 14 individual muscles of senior male sprinters. The number of axial images slices used to assess the T2 of each muscle was averaged across all participants. T2 value data are presented as mean (SD), with individual muscle measurements being average of multi slices regions of interest (ROIs).

	Pre			Post			*p* value	Cohen’s *d*
	Mean	SD	Range		SD	Range		
***T2 value, ms***								
rectus abdominis	33.09	(0.82)	31.27–33.90	34.25	(1.97)	31.93–38.38	0.096	0.76
lateral abdominals	34.57	(0.81)	33.24–35.64	40.68^*^	(2.58)	38.10–45.45	0.000	3.19
psoas major	34.12	(1.17)	32.18–36.26	38.55^*^	(2.66)	35.87–44.75	0.000	2.15
iliac	33.94	(0.84)	32.62–35.44	36.80	(2.78)	33.78–43.95	0.010	1.39
erector spinae	34.79	(1.06)	33.28–36.45	39.51^*^	(1.96)	37.00–43.48	0.000	2.99
gluteus maximus	35.95	(1.09)	34.21–37.63	43.04^*^	(2.63)	39.33–47.96	0.000	3.52
gluteus medius	35.27	(1.18)	33.68–37.25	37.75^*^	(1.66)	34.73–39.90	0.003	1.73
gluteus minimus	35.35	(1.48)	33.10–37.43	36.05	(0.99)	34.37–37.32	0.106	0.55
obturator internus	34.61	(1.52)	32.25–37.30	36.17	(2.24)	32.37–39.20	0.075	0.82
piriformis	35.21	(1.63)	33.40–38.83	37.95	(2.84)	35.25–43.95	0.014	1.18
rectus femoris	32.75	(1.16)	31.13–35.05	35.26^*^	(1.41)	32.87–37.10	0.000	1.94
tensor fasciae latae	32.18	(1.37)	29.43–34.35	35.72^*^	(2.16)	31.93–39.18	0.000	1.96
sartorius	32.05	(0.58)	31.45–33.32	35.72^*^	(3.29)	31.73–43.84	0.003	1.55
pectineus	35.45	(2.19)	32.73–40.00	42.13^*^	(2.25)	39.00–46.70	0.000	3.01

* denotes Pre < Post, p < 0.05 (Bonferroni collection, p < 0.004); SD, standard deviation.

RA, rectus abdominis; LA, lateral abdominals; IL, iliac; Gminimus, gluteus minimus;

PI, piriformis; SA, sartorius;TFL, tensor fasciae latae; RF, rectus femoris; PE, pectineus

Table 3 shows the percent change in T2 values before and after the 60-m round-trip sprint. There was a significant difference in the rate of change in T2 values between the muscles. The rate of change in T2 values was particularly high for the lateral abdominal, psoas major, erector spinae, gluteus maximus, and pectineus muscles compared with the other muscles.

**Table 3:** Rate of change in T2 value after sprinting. The rate of change was calculated by dividing the Pre value by the Post value (%).

	Change of T2 (%)		Statistically lower than
	Mean	SD	
***T2 value, ms***			
rectus abdominis	3.13	(5.17)	
lateral abdominals	14.81	(4.21)	RF, SA, TFL, IL, RA, OI, G_minimus_
psoas major	11.19	(5.47)	SA, TFL, RA
iliac	7.37	(6.29)	SA
erector spinae	11.76	(4.68)	RF, SA, TFL, RA
gluteus maximus	16.24	(4.62)	without LA, PE
gluteus medius	6.41	(5.16)	RF, TFL, RA
gluteus minimus	1.93	(3.36)	
obturator internus	4.04	(6.70)	
piriformis	6.87	(6.34)	
rectus femoris	7.04	(3.45)	
tensor fasciae latae	9.77	(3.86)	
sartorius	9.73	(6.47)	
pectineus	15.74	(5.26)	RF, SA, TFL, IL, RA

Statistical significance was set at p < 0.05 (Bonferroni collection, p < 0.004). SD, standard deviation;

RA, rectus abdominis; LA, lateral abdominals; IL, iliac; Gminimus, gluteus minimus;

PI, piriformis; SA, sartorius; TFL, tensor fasciae latae; RF, rectus femoris; PE, pectineus

## Discussion

In this study, T2-weighted images were used to quantify trunk and pelvis muscle activity during sprinting. The magnitude of the effect of the change in T2 values after sprinting was medium to large for all muscles. However, there were differences in the levels of muscle activity among the muscles. These findings suggest that although many muscles in the trunk and pelvis region are activated during sprinting, the number of muscles with pronounced activity is limited, and targeted training of these muscles may improve sprinting.

In this study, the gluteus maximus had the highest percentage change in T2 values among the included muscles. The results are consistent with those of previous studies that identified lower extremity muscle activity levels in sprinting (Yoshimoto et al., 2022). Previous studies investigating the relationship between sprinting and muscle volume have also shown that sprinting performance is higher in individuals with a larger gluteus maximus (Miller et al., 2020; Sugisaki et al., 2018). This is because the hip extensors are the muscles that execute the backswing of the leg during the support phase (Wiemann and Tidow, 1995), and the gluteus maximus is a primary hip extensor (Floyd, 2020). As hip extensors contribute to horizontal translation during running (Morin et al., 2015), the gluteus maximus should be strongly activated to generate explosive propulsive forces. Therefore, we inferred that the gluteus maximus was significantly activated in this study.

The psoas major involved in hip flexion also exhibited high muscle activity (Floyd, 2020). The psoas major and rectus femoris muscles have been of focus in hip flexion during sprinting (Copaver et al., 2012; Ema et al., 2018). Previous studies have shown that the psoas major is crucial in sprinting (Copaver et al., 2012; Hoshikawa et al., 2006). Sprinters have a larger psoas major than non-sprinters (Handsfield et al., 2017), and those with higher sprinting performance have a larger psoas major (Copaver et al., 2012; Sugisaki et al., 2018; Tottori et al., 2021). Theoretically, sprint acceleration performance depends on maximal horizontal power (P_max_) (Samozino et al., 2021), and there is a significant correlation between this performance and the iliopsoas (Bellinger et al., 2021). The iliopsoas comprises the psoas major and the iliac. In this study, high muscle activity was observed in the psoas major, which may be vital during acceleration.

Hip flexion torque is also observed from the latter half of the support phase to just after the takeoff in sprinting (Baba et al., 2000), and hip flexion torque during the swing phase is significantly associated with sprint velocity (Ema et al., 2018). Considering the results of this study and previous studies, the psoas major contributed to sprint performance, including acceleration performance (P_max_), by exerting hip flexion torque around the takeoff during sprinting. In this study, the pectineus also exhibited high muscle activity during sprinting. Similarly to the psoas major and rectus femoris muscles, the pectineus is involved in hip flexion (Floyd, 2020). As mentioned earlier, hip flexion is an essential joint movement in sprinting, and the pectineus may be strongly involved in this movement. The results of this study provide new insights, as no previous reports have focused on the activity of the pectineus in sprinting. However, future studies should examine the levels of muscle activity and size of the pectineus as well as the effects of training on pectineus growth.

In contrast to the present results, it was previously shown that the T2 values of the rectus femoris did not significantly change after sprinting using a protocol similar to that used in the present study (Yoshimoto et al., 2022). The discrepancy between the two studies may be attributed to the small contribution of the rectus femoris. However, the lack of significant T2 changes in the rectus femoris muscle in the previous study could be attributed to differences in the location of the tested muscle. The previous study used one slice of the muscle belly; however, this study used multiple proximal slices. Furthermore, differences in muscle activity levels could be observed based on the position from which the data were collected, and this point needs to be investigated in future studies.

The trunk muscles of the external obliques, lower abdomen, and lumbosacral erector spinae are active during submaximal running (Behm et al., 2009). However, there are no reports on maximal sprinting. In this study, high muscle activity was observed in the lateral abdominal muscles and erector spinae during maximal sprinting. Previous studies on trunk movement during running have shown that accumulated fatigue leads to an increased trunk forward lean (Apte et al., 2021). Additionally, trunk flexion is associated with a significant decrease in step length and an increase in stride frequency (Warrener et al., 2021). In other words, a forward trunk lean negatively affects running performance, and it is possible that during running, the spine generates extensor force to counteract this. Moreover, it has been revealed that as the running speed increases, the range of motion in trunk rotation also increases (Lang et al., 2023). In sprinting, where higher speeds are achieved, this movement may be even more pronounced. Therefore, spinal extension and rotation are essential elements in sprinting, and the erector spinae and lateral abdominal muscles may be strongly activated to facilitate these movements. In addition, it is possible that trunk muscles are isometrically active for stability. The T2 value measurements in this study could not clarify which sprinting movements were active. Therefore, it is necessary to verify which trunk muscles are active in the future.

## Limitations

In terms of the limitations of this study, we did not examine factors such as kinematics, kinetics, and electromyography during sprinting. In the sprint, information about the functional role of each muscle is essential, and will be a matter for future studies. In addition, the participants were male athletes who routinely performed sprint training; therefore, it is unclear whether similar muscle activity may be seen in athletes from other sports, non-athletes, female athletes or children. Previous studies have shown differences in muscle volume and a cross-sectional area according to the sports level and sex (Hoshikawa et al., 2006; Miller et al., 2020). There are also sex differences in the effect of the muscle cross-sectional area on sprinting performance (Hoshikawa et al., 2006). Therefore, sprint muscle activity differs depending on factors such as the performance level, sport, sex, and age. Further investigations are required to study these topics.

## Perspective

The results of this study show that the gluteus maximus acts as a hip extensor, the psoas major and pectineus maximus act as hip flexors, and the lateral abdominals and erector spinae act as trunk stabilizers during sprinting. These results suggest that the enhancement of these muscles may improve sprinting performance. Several training programs improve these muscles (Andersen et al., 2018; Liebenson et al., 2009; Sarti et al., 1996). A deadlift and a hip thrust (Andersen et al., 2018) are exercises that increase gluteus maximus and erector spinae muscle activity. The curl-up also produces higher muscle activity than running at 80% VO_2max_ in the external abdominal oblique muscles (Behm et al., 2009). These exercises could improve the activation of muscle groups involved in sprinting, as identified in this study. Previous studies have introduced training associated with the psoas major (Hu et al., 2011; Peltonen et al., 1998; Revel et al., 1993). In one study (Hu et al., 2011), muscle activity was observed in the psoas major and iliac muscles during leg raises, and higher muscle activity was observed when the weights were loaded. However, only one study has quantified the level of psoas major muscle activity, and the type of exercise that results in high muscle activity remains unclear. This is because the psoas muscle is located deep in the trunk, making it difficult to quantify muscle activity levels using surface EMG. In addition, no studies have focused on training to improve the pectineus muscle. The pectineus is not large, however, it is involved in hip flexion, adduction, and external rotation. These movements are assumed to be joint movements involved in sprinting, and the pectineus may strongly contribute to the execution of these movements. Therefore, future studies should identify training methods that can improve the performance of trunk and pelvis muscles, including the psoas major and the pectineus
